# Age-dependent defective TGF-beta1 signaling in patients undergoing coronary artery bypass grafting

**DOI:** 10.1186/1749-8090-9-24

**Published:** 2014-02-04

**Authors:** Santiago Redondo, Jorge Navarro-Dorado, Marta Ramajo, Úrsula Medina, Pedro Molina-Sanchez, Zaady Garces, Mauricio García-Alonso, Fernando Reguillo, Enrique Rodriguez, Vicente Andres, Teresa Tejerina

**Affiliations:** 1 Department of Pharmacology, School of Medicine, Universidad Complutense de Madrid, Av. Complutense s/n, 28040 Madrid, Spain; 2 Department of Molecular and Genetic Cardiovascular Pathophysiology, Spanish National Center for Cardiovascular Research (CNIC), Madrid, Spain; 3 Service of Cardiac Surgery, Hospital Clinico San Carlos, Madrid, Spain; 4 Service of General and Abdominal Surgery, Hospital Clinico San Carlos, Madrid, Spain

**Keywords:** Growth factors, Coronary disease, Aging, Surgery

## Abstract

**Background:**

Transforming growth factor beta (TGF-β1) is a pleiotropic cytokine, which is deregulated in atherosclerosis; however the role of age in this process is unknown. We aimed to assess whether TGF-β1 signaling is affected by age.

**Methods:**

Vascular smooth muscle cells (VSMC) were obtained from patients undergoing abdominal surgery. Levels of TGF-β1 were measured by ELISA in sera from 169 patients undergoing coronary artery bypass grafting (CABG). The p27 expression was determined by Western blot from internal mammary arteries (IMA) obtained from CABG patients (n = 13). In VSMC from these patients undergoing abdominal surgery, secretion of TGF-β1 was determined by ELISA of cell-conditioned media.

**Results:**

In VSMC from aged patients we observed a lower TGF-β1 secretion, measured as TGF-β1 concentration in cell conditioned medium *(p* < 0.001). This effect was correlated to an age-dependent decrease of p27 expression in IMA from aged CABG patients. In a similar manner, there was an age-dependent decrease of serum TGF-β1 levels in CABG patients (*p* = 0.0195).

**Conclusions:**

VSMC from aged patients showed a higher degree of cellular senescence and it was associated to a lower TGF-β1 secretion and signaling.

## Background

Atherosclerosis is the leading global cause of morbimortality [1]. TGF-β1 is a pleiotropic cytokine which is deregulated in atherosclerosis [2]. However, the role of aging in atherosclerotic-related TGF-β1 deregulation remains obscure. TGF-β1 signaling is complex and has many cross-talks with other signaling cascades [2]. One major common mediator of TGF-β1 signaling is p27, which links TGF-β and contact inhibition to cell cycle arrest [3,4].

The role of TGF-β1 is complex and cell-context dependent. In atherosclerosis, TGF-β1 is considered as a protective cytokine, which regulates vascular smooth muscle cell (VSMC) proliferation and thus decreases intima-media thickness [2,5]. It is also able to reduce vascular inflammation due to its inhibitory activity on plaque monocytes and T cells [6]. This atheroprotective effect is shared by its signaling protein p27, whose disruption has been shown to aggravate atherosclerotic lesions in animal models [7,8]. In human vascular samples, atherosclerotic lesions have been linked to reduced expression of p27 [9,10]. However, in advanced atherosclerosis TGF-β1 may behave as a proatherogenic substance by inducing enhanced fibrosis and increase of vascular tone, possibly by means of a defective signaling [2].

Many vascular phenomena which take part in atherosclerosis mimic what can be found in the process of vascular aging [11]. Of note, the role of human vascular aging in TGF-β1 is still an unexplored area. Moreover, we still do not have reliable methods to correlate chronological versus biological vascular age in the clinical arena. Therefore, we aimed to investigate the effect of age in human VSMC TGF-β1 secretion. In addition, we assessed p27 expression in a sample of internal mammary arteries (IMA) of 13 patients undergoing coronary artery bypass grafting (CABG). Eventually, we determined the pre-surgical serum levels of TGF-β1 in a group of 169 patients undergoing CABG.

## Methods

### Patients

For cell cultures, a group of patients was recruited from the service of Abdominal Surgery (Hospital Clinico San Carlos, Madrid, Spain). For the study of TGF-β1 in patients, two groups were recruited from those undergoing CABG at the Cardiac Surgery Service (Hospital Clinico San Carlos, Madrid, Spain). The first one (n = 36) was composed by patients where a remaining IMA segment was avaliable. From them, in 13 patients the arterial segment could yield enought protein to be assessed. The second group (n = 169) included patients with an available pre-surgical serum sample. In all cases patient data included: age, gender, active smoker, body mass index (BMI), cardiovascular risk factors, creatinine, leukocytes, monocytes and platelets. Clinical and analytical data of CABG patients from both groups are shown in the Tables 1 and 2, respectively. In both patient groups, exclusion criteria included inflammatory disease, renal and liver failure and cancer. The study was conducted according to the Declaration of Helsinki and approved by the local ethical committee (Ethical Committee, Hospital Clinico San Carlos, Madrid, Spain). Written informed consent was obtained from all patients.

**Table 1:** Clinical and analytical parameters from patients undergoing CABG whose IMA were used for the study

	**<55**	**55-64**	**65-74**	**>75**	**P**
Number	4	10	14	8	
Age	41.25	59.90	69.43	80.63	P < 0.0001*
Sex (% men)	4 (100%)	9 (90%)	13 (92.86%)	6 (75%)	0.5129^†^
BMI	26.99 ± 1.883	26.85 ± 0.8519	27.14 ± 0.641	27.67 ± 0.960	0.9396*
EF	57.5 ± 3.379	53.60 ± 5.494	57.36 ± 4.514	60.38 ± 3.407	0.8181*
Tobacco	0 (0%)	5 (50%)	2 (14.28%)	0 (0%)	0.0281^†^
Alcohol	0 (0%)	1 (10%)	0 (0%)	0 (0%)	0.4446^†^
Hypertension	3 (75%)	5 (50%)	9 (64.28%)	6 (75%)	0.6871^†^
Dyslipemia	1 (25%)	5 (50%)	6 (42.86%)	5 (62.5%)	0.6414^†^
DM	0 (0%)	3 (30%)	5 (35.71%)	4 (50%)	0.3786^†^
IM < 30 days	1 (25%)	5 (50%)	5 (35.71%)	3 (37.50%)	0.7676^†^
Creatinine	0.9925 ± 0.1291	0.9400 ± 0.0718	1.065 ± 0.0787	1.030 ± 1.004	0.7326*
Leukocytes	7800 ± 1476	7670 ± 606.3	7107 ± 301.2	7375 ± 854.6	0.8710*
Monocytes	875 ± 232.3	730 ± 109.6	600.0 ± 43.22	600.0 ± 73.19	0.2599*
Platelets	244000 ± 38899	236800 ± 27110	216357 ± 12395	183625 ± 2002	0.2949*

*One-way ANOVA. ^†^Chi-squared test.

BMI: body mass index. EF: Ejection fraction. DM: diabetes mellitus. IM < 30 days: myocardial infarction within 30 days before surgery.

**Table 2:** Clinical and analytical parameters from patients undergoing CABG whose pre-surgical sera were used for the study

	**<55**	**55-64**	**65-74**	**>75**	**P**
Number	23	48	61	31	
Age	49.61	59.75	69.08	76.77	P < 0.0001*
Sex (% men)	23 (100%)	42 (87.5%)	52 (85.24%)	17 (56.67%)	0.0002^†^
BMI	26.70 ± 0.643	27.58 ± 0.504	27.60 ± 0.458	27.13 ± 0.645	0.8639*
EF	58.52 ± 3.311	59.83 ± 2.000	59.95 ± 1.822	59.71 ± 2.049	0.4084*
Tobacco	10 (43.48%)	13 (27.08%)	12 (17.64%)	4 (12.90%)	0.0065^†^
Alcohol	13 (56.52%)	29 (60.42%)	33 (54.10%)	6 (12.90%)	0.0023^†^
Hypertension	7 (30.43%)	22 (45.83%)	40 (65.57%)	20 (64.52%)	0.012^†^
Dyslipemia	12 (52.17%)	30 (62.50%)	27 (44.26%)	16 (51.61%)	0.2871*
DM	5 (23.74%)	18 (37.50%)	20 (32.79%)	17 (54.84%)	0.0958^†^
IM < 30 days	11 (47.83%)	24 (50%)	35 (57.38%)	16 (51.61%)	0.7676^†^
Creatinine	1.030 ± 0.027	1.060 ± 0.024	1.130 ± 0.027	1.131 ± 0.045	0.0765*
Leukocytes	7854 ± 343.3	7592 ± 272	7466 ± 250.8	7120 ± 406.5	0.5640*
Monocytes	635.7 ± 41.40	612.6 ± 33.45	600.0 ± 24.63	617.7 ± 33.16	0.9078*
Platelets	227000 ± 12379	241128 ± 10477	224164 ± 7085	234452 ± 14260	0.5895*
TGF-β	42.21 ± 2.715	41.87 ± 2.187	36.44 ± 1.669	33.35 ± 2.092	0.0157*
TGF-β/platelet*1000	0.1937 ± 0.0129	0.1842 ± 0.0121	0.1684 ± 0.008	0.1583 ± 0.013	0.2220*

*One-way ANOVA. ^†^Chi-squared test.

BMI: body mass index. EF: Ejection fraction. DM: diabetes mellitus. IM < 30 days: myocardial infarction within 30 days before surgery.

### Cell cultures

Mesenteric arteries from patients undergoing abdominal surgery were used for VSMC culture. The arteries were extracted during the surgical procedure and immediately kept in RPMI medium (Life-Technologies, Barcelona, Spain) at 4°C. Whithin the next 3 h, they were mechanically disrupted in and the extracellular matrix was digested using collagenase at 4.5% for 3 h at 37°C. Cells were maintained in RPMI containing 10% foetal calf serum (FCS) and 1% antibiotic-antimycotic (Life-Technologies, Barcelona, Spain). The cells exhibited typical “hill and valley” morphology. Vascular smooth muscle cell phenotype was assessed as positivity for smooth muscle α-actin by both Western blot and confocal microscop (data not shown). Experiments were performed between passages 3 and 5. Primary cell cultures were maintained for 3 weeks in drug-free cell culture medium before a viable cell line could be obtained.

### Extraction of human arteries

Internal mammary arteries (IMA) were collected by the surgeons during the surgical procedure (Hospital Clinico San Carlos, Madrid, Spain). They were immediately kept in RPMI medium at 4°C within the next few minutes. They were then kept in carbonic ice at -70°C.

### Assessment of TGF-β1 concentration by ELISA

In cell culture experiments, VSMC obtained from mesenteric arteries were cultured in 24-well plates, serum free-cell conditioned media were extracted at 48 h and frozen at -70°C. Protein concentration was calculated using the bicinchionic acid assay. In patients, blood was centrifuged at 1500 rpm for 15 min; serum was extracted and frozen at -70°C. In both cases, total levels of TGF-β1 (active plus acid-activatable) were assessed by ELISA (R&D Systems, Minneapolis, MN, USA). This kit proved highly reproducible in a large comparative study of several ELISA kits for TGF-β [12]. For the TGF-β1 levels from cell conditioned media, data are expressed as pg/mg protein,

### Western blotting

We measured the expression of p27 in IMA from CABG patients. Tissues from IMA were disrupted with TissueLyser LT apparatus (Qiagen, Chatsworth, California, CA, USA) in ice-cold lysis buffer containing 50 mM Tris–HCl pH 7.5, 1% (w/v) Triton X-100, 150 mM NaCl and 1 mM DTT, supplemented with phosphatase and protease inhibitors (Roche Applied Science, Indianapolis, IN, USA). Lysates were centrifuged at 14000 rpm for 20 min (4°C) and protein concentration in the supernatants was determined using the Bradford assay (BioRad Laboratories. Richmond, CA, USA). Proteins (25 μg) were separated onto 12% SDS-polyacrylamide gels and Western blot analysis was carried out using anti-p27 (1/1000, 610242 BD Transduction Laboratories San Diego, CA, USA) and anti-ERK2 (1/1000, Santa Cruz Biotechnology, Santa Cruz, CA, USA) antibodies. A similar procedure was performed for Smad2 and Smad3, as well as their phosphorylated forms (Sigma, Madrid, Spain). Relative protein level was determined by densitometry using the ImageQuant software (Amersham, Uppsala, Sweden).

### Statistical analysis

The results are expressed as the mean±SEM (standard error mean). A statistical analysis of the data was carried out by a Student’s t test or by a one-way ANOVA when necessary. Correlations were compared using Pearson’s correlation test, whereas categorical variables were compared using Chi-squared test. Differences with a *p* value of less than 0.05 were considered statistically significant.

## Results

### Age-dependent defective TGF-β1 secretion in human VSMC

**Figure 1:**
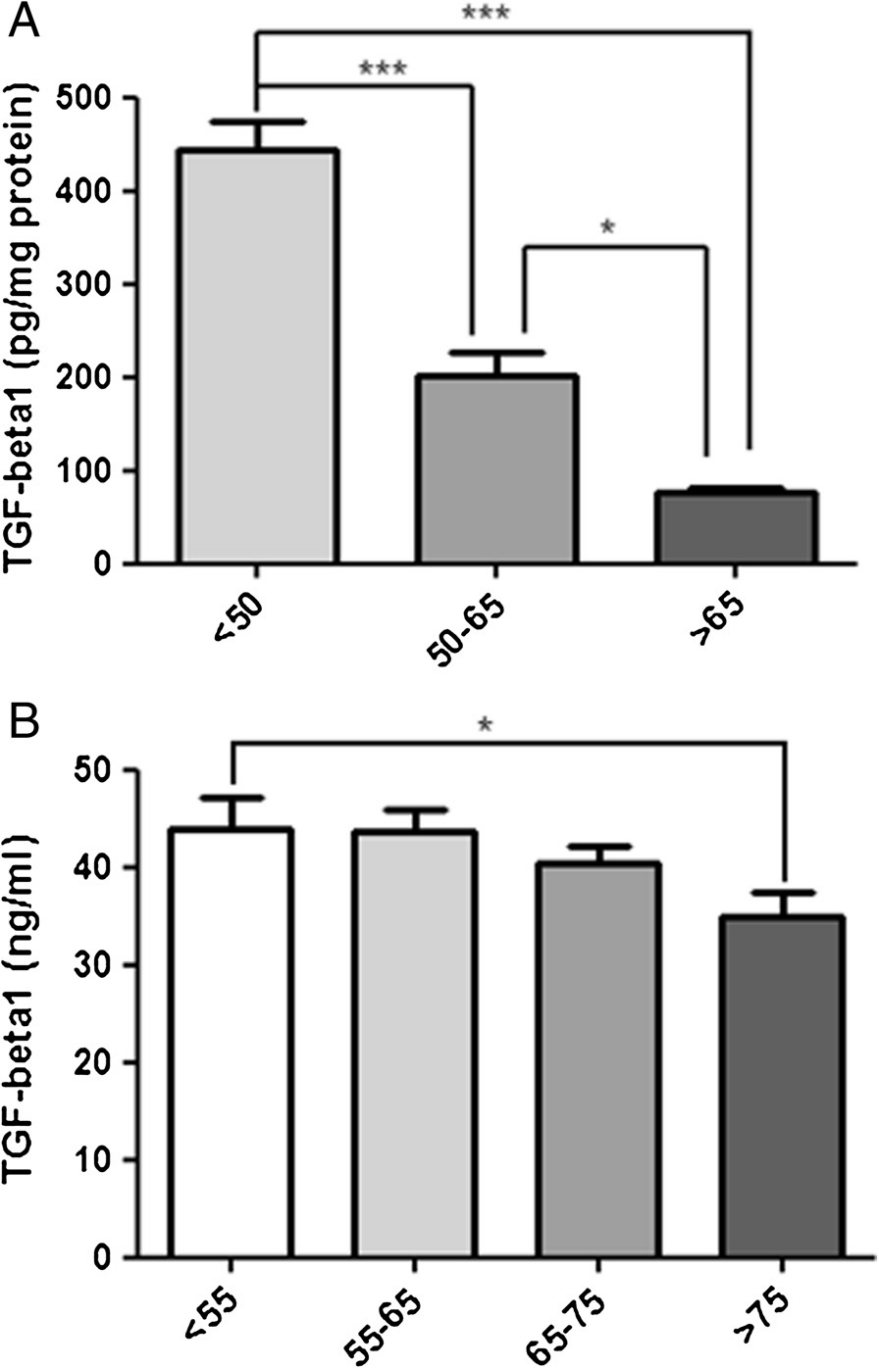
**Age-dependent TGF-β1 concentration.** Panel **A**: Cells were cultured in plates and conditioned media were collected. Panel **B**: Concentration of TGF-β1 was assessed in the pre-surgical serum of CABG patients according to Methods. *p < 0.05, ***p < 0.001.

We aimed to assess whether TGF-β1 secretion was influenced by age. As shown in Figure 1A, human VSMC exhibited a progressive age-dependent reduction in the secretion of TGF-β1, as revealed by analysis of cell conditioned media. Of note, this decrease was statistically significant between < 50 and 50–65 years old, and reached the strongest effect in VSMC from patients aged > 65 years old.

### Age-dependent decrease of TGF- β1 in serum of patients

We next tried to assess whether CABG patients, with a strong burden of angiographically demonstrated atherosclerotic vascular disease, had lower levels of serum TGF-β1 signaling related to aging. Therefore, we examined two different groups of patients who underwent CABG. The first group was composed by 36 patients where remaining IMA was available for analysis. As shown in Table 1, in this group only an inverse association with smoking was associated with age. However, in the second group, composed by CABG patients whose pre-surgical sera were available (n = 169), old age was associated to higher percentage of women and hypertension rate, whereas it was inversely related to smoking, drinking alcohol and serum levels of TGF-β1 (Table 2). As shown in Figure 1B, the concentration of TGF-β1 in the pre-surgical sera of CABG patients was decreased in the oldest age group in a significant manner. However, TGF-β1 levels did not vary according to other clinical parameters, such as pharmacological treatments in the multivariate analysis (data not shown).

### Age-dependent reduction in p27 expression in IMA from patients

**Figure 2:**
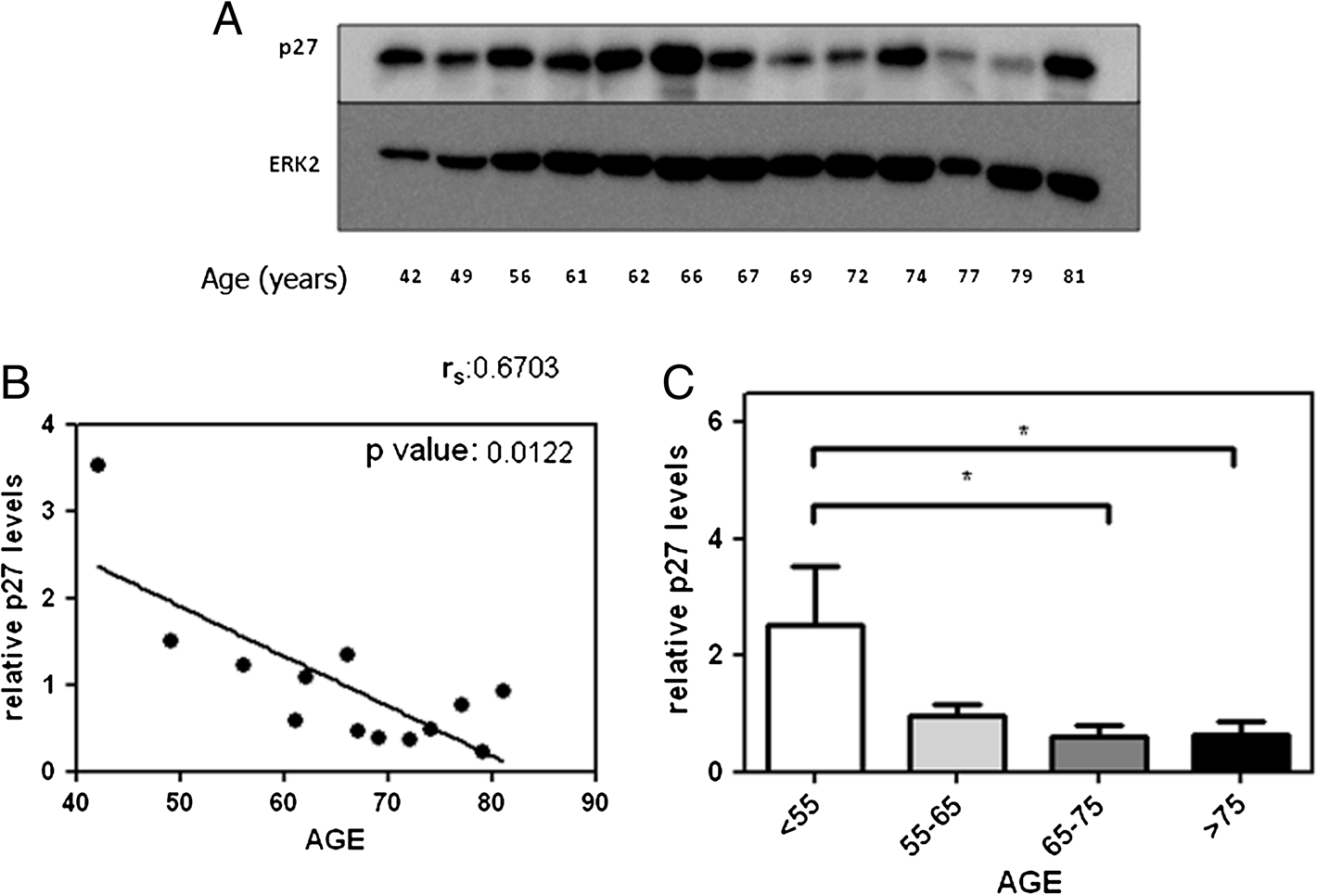
**Age-dependent decreased p27 in IMA from CABG patients.** Panel **A**: Representative Western blot of p27 expression *versus* ERK in IMA from CABG patients. Panel **B**: p27 expression and age show a significant inverse correlation. Panel **C**: age-dependent p27 decrease remains statistically significant when compared among several age groups. *p < 0.05.

**Figure 3:**
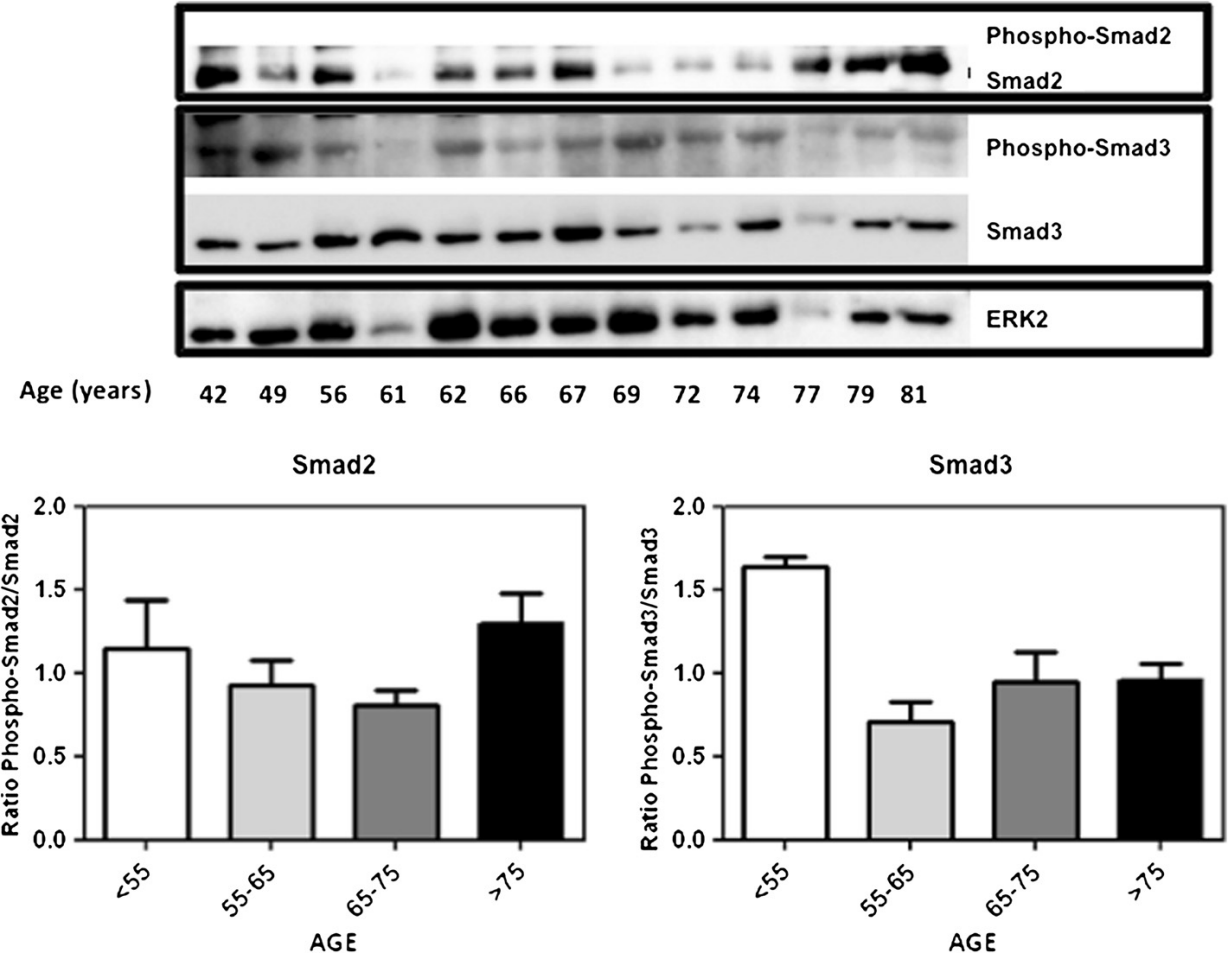
**Effect of age on Smad2 and Smad3 phosphorylation.** Top dot blots show the expression of Smad2 and Smad3 as well as their phosphorylated forms. Bottom bars show quantifications among different age groups. No significant differences were found.

Having demonstrated that TGB-β1 is reduced with aging, we examined in IMA from CABG patients the expression of p27, which is a direct marker of TGF-β1 signaling [4]. Western blot analysis revealed a statistically significant decrease of p27 expression during aging (Figure 2A, B and C). This reinforces the idea that advanced age is associated to decreased TGF-β1 signaling in the human vascular wall when measured as p27 expression, similar to what found in atherosclerotic lesions [8]. However, when phosphorylation of Smad2 and Smad3 were assessed, no significant age-related differences were noted among different age groups (Figure 3).

### Correlations among serum TGF-β1, age and platelets in CABG patients

**Figure 4:**
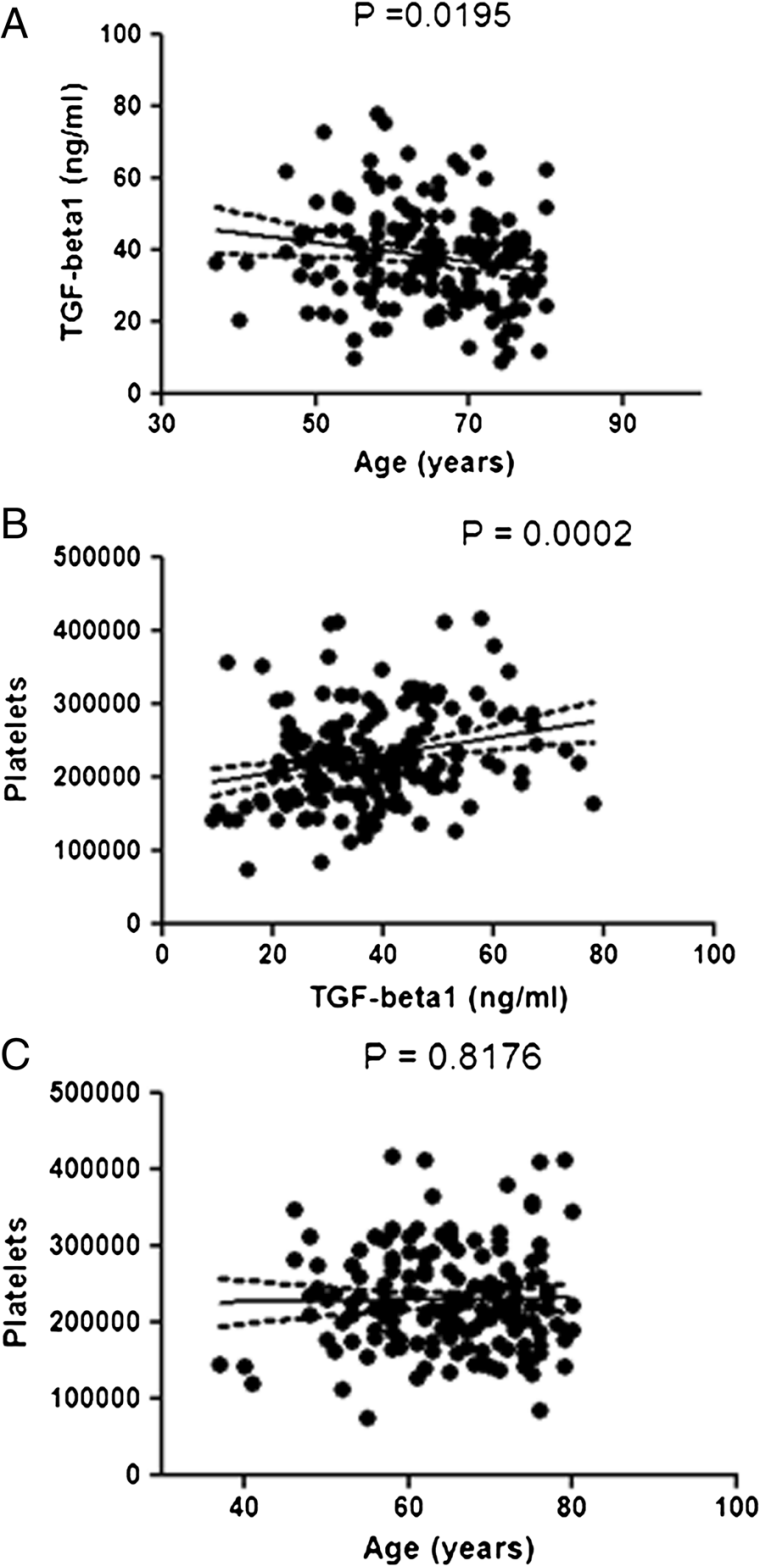
**Correlations among TGF-β1, age and platelets.** Panel **A**: Correlation between pre-surgical serum levels of TGF-β1 and age. Panel **B**: Correlation between pre-surgical serum levels of TGF-β1 and the number of platelets of the CABG cohort. Panel **C**: Correlation between the number of platelets and age. Pearson’s correlation tests were run according to Methods.

Since it is widely accepted that a major amount of serum TGF-β1 comes from platelets [2] and an age-dependent platelet decrease has been found in healthy populations [13], we tried to assess whether age-dependent serum levels of TGF-β1 were affected by platelet number. First, a correlation graph was built for TGF-β1 and age. Yet again, this inverse correlation was statistically significant (Figure 4A). Moreover, we found a strong positive correlation between serum TGF-β1 and platelets (Figure 4B). However, the comparison between age and platelets could not yield a significant association (Figure 4C). Thus, the age-dependent decrease in serum TGF-β1 in CABG patients is not a direct consequence of age-related platelet decrease. This finding is reinforced by the fact that serum TGF-β1 per platelet remained unchanged among age groups (Table 2).

## Discussion

The present study shows that advanced age implies a decrease of TGF-β1 secretion by human VSMC. This age-dependent TGF-β1 defect is further reproduced in CABG patients, where an age-dependent defective p27 expression is found at the vascular level, and pre-surgical serum concentrations of TGF-β1 are decreased in aged groups.

Despite that many vascular phenomena found in atherosclerosis are similar to what found in vascular aging [11], and the fact that the majority of atherosclerotic patients belong to the elderly [1], the mechanisms underlying age-dependent atherosclerotic disease remain poorly understood. In atherosclerosis, TGF-β1 seems to lose its atheroprotective effects [2,5]. TGF-β1 exerts its wide variety of biological actions by means of very complex signaling pathways, some of which converge in the expression of the cell cycle regulatory protein p27 [3,4]. In particular, decreased p27 expression has been linked to atherosclerotic vascular disease in murine models [7,8]. This concept is reinforced by the fact that human atherosclerosis has been linked to decreased serum levels of TGF-β [14,15]. At the same time, in animal models atherosclerosis can be experimentally accelerated when TGF-β1 is inhibited [6]. However, in advanced atherosclerosis, TGF-β1 may behave as a proatherogenic stimulus by increasing extracellular matrix formation and fibrosis [16] and subsequent hypertensive organ damage [17] after a progressive loss of a proper signaling [18], what has been termed the double-edged sword hypothesis [2]. Thus, decreased TGF-β1 signaling and loss of p27 expression [10] might be considered as a hallmark of atherosclerosis.

However, no data are available about the effect of age on TGF-β1 signaling pathway in humans. Our data clearly show that advanced age is strongly related to a lower TGF-β1 secretion in human VSMC conditioned medium (Figure 1A). This is in accordance to our finding of decreased serum levels of TGF-β1 in the oldest CABG patients (Figure 1B). Moreover, we noted a decreased p27 expression in human IMA from CABG patients (Figure 2A, B and C), although Smad2 and Smad3 phosphorylation was not affected in a significant manner (Figure 3). Eventually, this association was reinforced when a correlation was made between serum TGF-β1 and age in the entire cohort (Figure 4A). This age-dependent decrease of serum TGF-β1 in our group of CABG patients is in accordance with results in a Japanese population study [19], although the role of age in atherosclerosis-related TGF-β1 deregulation has not been evaluated to date. Given that the majority of serum TGF-β1 is secreted by platelets [2] and an age-related platelet decrease have been noted [13] one could argue that platelets mediate age-dependent TGF-β1 decrease in our sample. Of note, cell secretion of this cytokine is a process strictly regulated and seems to be regulated in an independent manner of its mRNA transcription [2]. However, we found a strong correlation between serum TGF-β1 and platelets (Figure 4B), similar to described elsewhere [20], whereas a significant association between age and platelets was not noted in our cohort (Figure 4C), possibly related to the factor that all CABG patients are severely affected by atherosclerosis. This lack of association can be related to a direct decrease of TGF-β1 from human VSMC in our cell culture model (Figure 2A) and is reinforced by the fact that all age groups had similar serum TGF-β1 per platelet (Table 2). Beyond the major role of platelets, a minor, albeit significant, role for VSMC in serum TGF-β1 is coherent to the current scientific evidence [21].

Outcome of CABG is highly variable among patients. Several risk scores have been established, the euroSCORE II (which includesx age) being one of the most important ones [22]. However, many other unknown risk factors influence CABG outcome [23]. Among them, the TGF-β1 pathway is implicated in atherosclerosis and cardiovascular drug response [24]. Adding a measurement of TGF-β1 function may complement current clinical risk scores and thus help to discriminate the best clinical option for every single patient, what can be regarded as a major clinical need [25]. In addition, correlation of the TGF-β1 pathway and the amount of cardiovascular disease will be the subject of coming research efforts.

## Conclusions

In conclusion, the present study show that decreased TGF-β1 signaling and concentrations may be considered as a hallmark of vascular aging in atherosclerotic patients. Thus, the assessment of serum levels of the TGF-β1 and its signaling in surgical samples may become a potential tool to measure vascular aging in the clinical arena.

## Abbreviations

TGF: Transforming growth factor; VSMC: Vascular smooth muscle cells; CABG: Coronary artery bypass grafting; IMA: Internal mammary artery; BMI: Body mass index; FCS: Fetal calf serum; ELISA: Enzyme-linked Immunoabsorbent assay; SEM: Standard error mean; ANOVA: Analysis of variance.

## Competing interests

The authors declare that they have no competing interests.

## Authors’ contributions

SR, VA and TT conceived the idea. SR, JN-D, MR, UM and PM-S performed the experiments. ZG, MA, FR and ER collected and analyzed the clinical data. SR, VA and TT wrote the manuscript. All authors read and approved the final version of the manuscript.
